# Premenstrual syndrome, premenstrual dysphoric disorder, and coping strategies in women with menstrual migraine

**DOI:** 10.3389/fneur.2026.1853733

**Published:** 2026-05-29

**Authors:** Alshimaa S. Othman, Mona Hussein, Alaa Elmazny, Salsabil Abo Al-Azayem, Azza Elashiry, Mennat-Allah Tarek, Heba Qassim Shamardal, Sara El-Sayed Abd El-Ghani, Mohamed Wagdy, Eman Hany Elsebaie, Doaa Lotfy Abd El Baky, Rehab Magdy

**Affiliations:** 1 Department of Neurology, Kasr-Alainy Faculty of Medicine, Cairo University, Cairo, Egypt; 2 Department of Neurology, Beni-Suef University, Beni-Suef, Egypt; 3 College of Medicine and Health Sciences, Manama, Bahrain; 4 Family Medicine Department, Faculty of Medicine, Fayoum University, Fayoum, Egypt; 5 Department of Psychiatry, Faculty of Medicine, Cairo University, Cairo, Egypt; 6 Department of Obstetrics and Gynecology, Beni Suef University, Beni Suef, Egypt; 7 Clinical Hematology Unit, Internal Medicine Department, Faculty of Medicine, Cairo University, Cairo, Egypt; 8 Faculty of Medicine, Modern University for Technology and Information, Cairo, Egypt; 9 Department of Public Health and Community Medicine, Kasr-Alainy Faculty of Medicine, Cairo University, Cairo, Egypt; 10 Department of Anesthesiology, Faculty of Medicine, Fayoum University, Fayoum, Egypt

**Keywords:** menstrual migraine, premenstrual syndrome, Premenstrual Dysphoric Disorder (PMDD), premenstrual coping strategies, women-diseases

## Abstract

**Background and objectives:**

Despite the considerable prevalence of premenstrual syndrome (PMS) in women with migraine, little attention has been given to the applied premenstrual coping strategies and their impact on headache severity, especially in menstrual migraine (MM). Therefore, this study aimed to investigate the severity of PMS, the Premenstrual Dysphoric Disorder (PMDD), the coping strategies used among women with MM versus those with non-MM, and to determine their impact on their perceived headache burden.

**Methods:**

In this cross-sectional study, existing diary data covering at least three consecutive menstrual cycles were reviewed to classify participants as having MM or non-MM. All patients were evaluated by the Headache Impact Test-6 (HIT-6), Premenstrual Symptoms Screening Tool (PSST) and the Premenstrual Coping Measure.

**Results:**

This study was conducted on 445 females with MM and 156 with non-MM. Females with MM had significantly higher PSST and HIT-6 scores (*p* = 0.001, 0.002, respectively) than the non-MM group. The diagnosis of PMDD was confirmed in 5.6 and 1.9% of females with and without MM, respectively (*p* = 0.06). By linear regression analysis, the presence of PMDD was associated with an increase of 3.113 points in HIT-6 scores (95%CI: 0.607–5.915, *p* = 0.016). In addition, higher PSST scores and lower awareness and acceptance scores were significantly associated with higher HIT-6 scores (*p* ≤ 0.001 for both).

**Conclusion:**

Women with a confirmed diagnosis of PMDD, high PMS symptom severity and lower frequency of using the awareness and acceptance coping strategy are at risk of increased headache burden.

## Introduction

Premenstrual syndrome (PMS) refers to a cyclic occurrence of physical, psychological, and cognitive symptoms during the luteal phase of the menstrual cycle that resolves shortly after the onset of menstruation, affecting about 48–70% of menstruating women (1, 2). The luteal phase symptoms are merely worrisome in about 40% of women; nevertheless, they do not need treatment. However, 25% had bothersome symptoms that do not impact functioning; while the symptoms are severe in 10–15%, which may significantly impair their daily life (3). PMDD (Premenstrual Dysphoric Disorder) is a severe form of PMS that encompasses extreme emotional disturbances, depression, intense irritability, or rage that is most often severe and debilitating (4).

The relationship between menstrual migraine (MM) and PMS has been explored through diary-based studies (5). They found that in women with MM, headache intensity was significantly associated with both physical and psychological symptom scores of PMS.

The prior studies have suggested that women devote a range of premenstrual coping strategies so as to master and reduce PMS (6). If a woman resorts to maladaptive coping strategies, she may experience a more negative mood and perceive more severe changes in her physical status (7). However, no available studies had addressed this issue in women with migraine. Determining PMS experience and coping mechanisms among women with migraine can contribute to the development of efficient strategies.

Thereby, this research is carried out to determine the severity of PMS, PMDD and coping experiences among women with MM versus those with non-MM. A second aim was to investigate how the severity of PMS and the type of applied premenstrual coping strategies are related to the perceived headache burden.

## Methods

### Study design, setting, and participants

This cross-sectional study was carried out between October 2025 and February 2026 at three headache clinics in Egypt: Cairo, El-Fayoum and Beni-Suef University Hospitals. Women aged 18 to 45 years with a confirmed diagnosis of migraine according to the International Classification of Headache Disorders, 3rd edition (ICHD-3) (8), and available headache and menstrual diary records covering at least three consecutive menstrual cycles were eligible for inclusion.

Patients were excluded if they had used hormonal treatments, migraine preventive medications, antidepressants, or supplements commonly taken for premenstrual symptoms (such as evening primrose) in the 3 months prior to enrollment. Additional exclusion criteria included pregnancy, lactation, irregular menstrual cycles that could interfere with accurate classification of menstrual migraine, and participants with missing data or those who refused or were unable to complete the study questionnaires.

### Baseline clinical and menstrual characteristics

Demographic and clinical data, including participants’ age, duration of headache history, and detailed menstrual characteristics (cycle regularity, average cycle length, and duration of menstrual bleeding), were recorded.

As part of our routine headache clinic practice, patients are instructed to maintain headache diaries and menstrual cycle tracking when a menstrual association is suspected. Existing diary data covering at least three consecutive menstrual cycles were reviewed and used to classify participants as having pure menstrual migraine / menstrually-related migraine or non-menstrual migraine (nMM), consistent with the criteria of ICHD-3 (8). To make it easier, both diagnoses of pure menstrual migraine/menstrually-related migraine were encompassed under a unified title of menstrual migraine.

### Headache-related disability

Headache-related disability was assessed using the Headache Impact Test–6 (HIT-6) (9), a validated six-item questionnaire that evaluates the impact of headaches on daily functioning. Each item is scored on a 5-point scale, with total scores ranging from 36 to 78, where higher scores indicate greater disability.

### Premenstrual symptoms assessment

Premenstrual symptoms were assessed using the Premenstrual Symptoms Screening Tool (PSST) (10), a 19-item self-report questionnaire designed to identify and differentiate PMS and PMDD. The tool evaluates both symptom severity and functional impairment.

### Coping strategies assessment

Coping strategies related to premenstrual changes were assessed using the Premenstrual Coping Measure (PMCM) (11), a validated 32-item scale evaluating domains such as Avoiding Harm, Awareness and Acceptance, Adjusting Energy, Self-Care, and Communication. Higher scores indicate greater utilization of coping strategies.

### Ethical considerations

The study was conducted in accordance with the Declaration of Helsinki. Ethical approval was obtained from the Fayoum University Ethics Committee (Approval No 809). Written informed consent was obtained from all participants prior to enrollment.

### Sample size calculation

The sample size was calculated before starting the study using G*Power version 3.1.9.7 Software. The effect size was calculated based on the mean values of HIT-6 scores in menstrual vs. non-menstrual migraine in a previous study (5). The statistical test used was the independent sample t-test. The type of power analysis was: *A priori*: compute required sample size- given *α*, power, and effect size. The input parameters were: tail (s) = two, effect size d = 0.397, α err prob. = 0.05, and power (1-*β* err prob) = 0.80. The output parameters were: noncentrality parameter *δ* = 2.819, critical *t* = 1.972, and Df = 200. A total sample size of at least 101 in each group was required to achieve a statistical power (1–β) 80%.

### Statistical methods

Data were analyzed using SPSS version 25 (IBM Corp., Armonk, NY, USA). The Kolmogorov–Smirnov test was used to test the normality of data. Quantitative data such as age, age at onset, duration of menstrual period, menstrual cycle length, MHD, PSST, PMCM, and HIT-6 scores were expressed as median and interquartile range. Categorical data, such as regularity of cycles and PMDD diagnosis, were expressed as a number and a percentage. The Mann–Whitney U-test was used to compare patients with MM and those with non-MM migraine in quantitative data, while the chi-square test was used to compare them in categorical data. Spearman correlation was used to correlate HIT-6 scores with MHD, PSST, and PMCM scores. Multiple linear regression analysis was done to identify factors associated with the HIT-6 total score after being adjusted for their potential mutual confounding effect. A stepwise regression method was employed to select the significant factors. The independent variables were determined based on their clinical relevance. *p* ≤ 0.05 was considered statistically significant. All tests were two-tailed.

## Results

### Demographic, menstrual, and clinical characteristics of patients with MM versus non-MM migraine

This study was conducted on 445 patients with MM migraine and 156 patients with non-MM migraine. Both groups were comparable regarding age (*p*-value = 0.605), duration of menstrual period (p-value = 0.400), menstrual cycle length (p-value = 0.191), regularity of cycles (*p*-value = 0.055), and PMDD diagnosis (*p*-value = 0.060).

However, patients with MM migraine had a significantly earlier age at onset than those with non-MM migraine (*p*-value ≤0.001) (Table 1).

**Table 1:** Compres menstraul migraine (MM) versus Non-menstraul migraine (non-MM).

Clinical characteristics		Patients with menstraul migraine (*n* = 445)	Patients with non-menstrual migraine (*n* = 156)	*p*-value
Age [median (IQR)]		35 (29–39)	35 (29–39)	0.605
Age at onset [median (IQR)]		22 (16–28)	25 (19–30)	< 0.001*
Duration of menstrual period [median (IQR)]		6 (5–7)	5 (5–7)	0.400
Menstrual cycle length [median (IQR)]		28 (25–28.5)	28 (26–29)	0.191
Regular cycles [*n* (%)]	Yes	385 (86.5%)	125 (80.1%)	0.055
	No	60 (13.5%)	31 (19.9%)	
PSST [median (IQR)]	Symptom items score	28 (22–33)	24 (16–31.75)	0.001*
	Functional items score	9 (6–11)	8.5 (5–10)	0.009*
	Total score	37 (29–43)	32 (22–41)	0.001*
PMDD Diagnosis [*n* (%)]	Yes	25 (5.6%)	3 (1.9%)	0.060
	No	420 (94.4%)	153 (98.1%)	
PMCM [median (IQR)]	Avoiding harm score	25 (20–29)	24 (19–29)	0.058
	Awareness and acceptance score	26 (22–32)	28 (21–33)	0.478
	Adjusting energy score	15 (11–17)	13.5 (10–16)	0.004*
	Self-care score	12 (8–14)	11 (7.25–13)	0.215
	Communicating score	12 (8–16)	10 (8–14)	0.002*
	Total score	89 (80–98)	85 (77.25–94)	0.001*
MHD [median (IQR)]		5 (3–9.5)	5 (3–10)	0.390
HIT-6 total score [median (IQR)]		63 (60–66)	62 (57–65)	0.002*

HIT-6, Headache Impact Test-6; MHD, Monthly headache days; PMCM, Premenstrual Coping Measure; PMDD, Premenstrual Dysphoric Disorder; PSST, Premenstrual Symptoms Screening Tool.

*Bold values indicate statistical significance, as *p* value < 0.05.

Regarding premenstrual symptoms, patients with MM had significantly higher PSST symptom items scores (*p*-value = 0.001), functional items scores (p-value = 0.009), and total scores (*p*-value = 0.001) compared with patients with non-MM (Table 1).

As for premenstrual coping, patients with MM had significantly higher premenstrual adjusting energy scores (*p*-value = 0.004) and communicating scores (p-value = 0.002). In contrast, no significant differences were observed between the two groups in avoiding harm (*p*-value = 0.058), awareness and acceptance (*p*-value = 0.478), or self-care scores (p-value = 0.215) (Table 1).

There was no statistically significant difference between patients with MM and those with non-MM migraine regarding MHD (p-value = 0.390), whereas patients with MM had significantly higher HIT-6 scores than those with non-MM migraine (p-value = 0.002) (Table 1).

The rank-biserial correlation for HIT-6 total score was 0.18 (95% CI: 0.07–0.29) and for PSST total score was 0.21 (95% CI: 0.10–0.31), indicating that the differences, while statistically significant, are also clinically meaningful.

### Demographic, menstrual, and clinical characteristics of patients with pure menstrual migraine versus those with menstrual-related migraine

Patients with pure menstrual migraine were significantly younger than those with menstrual-related migraine (*p*-value < 0.001) and had shorter menstrual cycles (*p*-value < 0.001), with a higher proportion reporting regular cycles (*p*-value = 0.046). While PSST scores and PMDD prevalence did not differ significantly, patients with pure menstrual migraine consistently demonstrated stronger coping strategies across all PMCM domains, including avoiding harm, self-care, and communicating (*p*-value < 0.001). Clinically, patients with pure menstrual migraine reported fewer MHD (p-value < 0.001) and lower HIT-6 total score (*p*-value < 0.001) (Table 2).

**Table 2:** Demographic, menstrual, and clinical characteristics of patients with pure menstrual migraine versus those with menstrual-related migraine.

Clinical characteristics		Patients with pure menstrual migraine (*n* = 130)	Patients with menstrual-related migraine (*n* = 315)	*p*-value
Age [median (IQR)]		33 (25.75–38)	37 (30–40)	<0.001*
Age at onset [median (IQR)]		24 (17–29)	20 (16–28)	0.091
Duration of menstrual period [median (IQR)]		5.5 (5–6)	6 (5–7)	0.393
Menstrual cycle length [median (IQR)]		26 (24–28)	28 (26–30)	<0.001*
Regular cycles [*n* (%)]	Yes	119 (91.5%)	266 (84.4%)	0.046*
	No	11 (8.5%)	49 (15.6%)	
PSST [median (IQR)]	Symptom items score	29 (23–33)	28 (21–34)	0.827
	Functional items score	10 (7–12)	9 (6–11)	0.073
	Total score	39 (31–43.25)	36 (28–43)	0.368
PMDD Diagnosis [*n* (%)]	Yes	4 (3.1%)	20 (6.3%)	0.165
	No	126 (96.9%)	295 (93.7%)	
PMCM [median (IQR)]	Avoiding harm score	27 (24–30.25)	24 (19–28)	<0.001*
	Awareness and acceptance score	25.5 (20–29)	27 (22–33)	0.026*
	Adjusting energy score	16 (13–18)	14 (11–17)	<0.001*
	Self-care score	13 (11–15)	10 (8–13)	<0.001*
	Communicating score	15 (10.75–18)	11 (8–15)	<0.001*
	Total score	97 (87–102)	87 (79–96)	<0.001*
MHD [median (IQR)]		4 (3–6)	5 (3–10)	<0.001*
HIT-6 total score [median (IQR)]		61 (58.5–64)	63 (60–67)	<0.001*

HIT-6, Headache Impact Test-6; MHD, Monthly headache days; PMCM, Premenstrual Coping Measure; PMDD, Premenstrual Dysphoric Disorder; PSST, Premenstrual Symptoms Screening Tool.

*Bold values indicate statistical significance, as *p* value < 0.05.

### Relationship of HIT-6 total score with premenstrual-related parameters

There were statistically significant positive correlations between HIT-6 scores and MHD (*r* = 0.284, *p*-value ≤ 0.001). Likewise, significant positive correlations were observed between HIT-6 scores and all PSST subscores, including symptom items scores (*r* = 0.276, *p*-value ≤0.001), functional items scores (*r* = 0.297, *p*-value ≤0.001), and total scores (*r* = 0.307, p-value ≤0.001) (Table 3).

**Table 3:** Correlations between HIT-6 total score and both PSST and PMCM scores.

Variables		HIT-6 total score	
		(*r*) coef.	*p*-value
MHD		0.284	< 0.001*
PSST	Symptom items score	0.276	< 0.001*
	Functional items score	0.297	<0.001*
	Total score	0.307	<0.001*
PMCM	Avoiding harm score	0.101	0.016*
	Awareness and acceptance score	−0.098	0.020*
	Adjusting energy score	0.149	< 0.001*
	Self-care score	0.041	0.327
	Communicating score	0.069	0.100
	Total score	0.093	0.025*

HIT-6, Headache Impact Test-6; MHD, Monthly headache days, PMCM: Premenstrual Coping Measure, PSST: Premenstrual Symptoms Screening Tool.

(*r*) Coef.: Spearman correlation coefficient.

*Bold values indicate statistical significance, as *p* value < 0.05.

Regarding PMCM, HIT-6 score showed statistically significant positive correlations with avoiding harm (*r* = 0.101, *p*-value = 0.016), adjusting energy (*r* = 0.149, p-value ≤0.001). In contrast, a significant negative correlation was observed with awareness and acceptance (*r* = −0.098, *p*-value = 0.020). No significant correlations were found between HIT-6 total scores and self-care (*r* = 0.041, *p*-value = 0.327) or communicating scores (*r* = 0.069, p-value = 0.100) (Table 3).

HIT-6 total score did not differ significantly according to menstrual cycle regularity (p-value = 0.238). In contrast, HIT-6 total score was significantly higher in patients with PMDD than in those without PMDD (*p*-value ≤ 0.001) (Table 4).

**Table 4:** HIT-6 total score in relation to regularity of the cycles and PMDD diagnosis.

Variables		HIT-6 total score [median (IQR)]
Regular cycles	Yes (*n* = 510)	63 (60–66)
	No (*n* = 91)	62 (58–66)
	P-value	0.238
PMDD diagnosis	Yes (*n* = 28)	66 (64.5–73.5)
	No (*n* = 573)	62 (59–66)
	*p*-value	< 0.001*

HIT-6, Headache Impact Test-6; PMDD: Premenstrual Dysphoric Disorder.

*Bold values indicate statistical significance, as *p* value < 0.05.

### Factors associated with HIT-6 score

Multiple linear regression analysis was done to identify factors associated with HIT-6 score. A stepwise regression method was employed to select significant factors, with PMDD, PSST, and PMCM subscores included as candidate independent variables.

The presence of PMDD was associated with an increase of 3.113 points in HIT-6 score (B = 3.261, 95% CI: 0.607–5.915, *p*-value = 0.016). In addition, higher PSST scores were significantly associated with higher HIT-6 score (B = 0.162, 95% CI: 0.113–0.212, p-value ≤ 0.001), whereas lower awareness and acceptance scores were independently associated with higher HIT-6 score (B = −0.139, 95% CI: −0.203 - -0.075, p-value ≤ 0.001) (Table 5).

**Table 5:** Factors associated with HIT-6 total score.

Independent predictors	*B*	*p*-value	95.0% CI for B		Collinearity statistics	
			Lower bound	Upper bound	Tolerance	VIF
PMDD	3.261	0.016*	0.607	5.915	0.901	1.110
PSST	0.162	<0.001*	0.113	0.212	0.900	1.112
Awareness and acceptance score	−0.139	<0.001*	−0.203	−0.075	0.998	1.002
(Constant)	60.489	0.000	58.075	62.902		

PMDD, Premenstrual Dysphoric Disorder; PSST, Premenstrual Symptoms Screening Tool.

*Bold values indicate statistical significance, as *p* value < 0.05.

## Discussion

The current study found that women with MM report more severe and disabling PMS relative to those without MM, posing a significant impact on their headache burden, as displayed by significantly higher HIT-6 scores.

On the contrary, Vetvik, MacGregor (5) found no significant differences between women with MM and those without MM with respect to PMS. Such a discrepancy could be explained partly by cultural differences, study designs, and the instrument used to assess PMS, as Vetvik, MacGregor (5) used an 11-item PMS questionnaire derived from the criteria of the DSM-IV for PMDD.

On the other hand, Vetvik, MacGregor (5) found a significantly higher HIT-6 score and MMDs among women with MM than those with non-MM, which aligned with the current findings.

Growing evidence supports the concept that estrogen helps boost serotonin production and reduces its degradation (12, 13). Therefore, a sharp decline in estrogen levels just before the menstrual cycle causes a corresponding fall in serotonin levels and a subsequent activation of the trigeminovascular system (14). This might explain the link between PMS and MM on the one hand, and the significant associations observed between HIT-6 cores and PSST scores on the other hand.

From the perspective of PMDD, the diagnosis was confirmed in 5.6 and 1.9% of females with and without MM, respectively. Notably, the former was significantly higher than the prevalence of PMDD in the general population of women of reproductive age (1.6%) as estimated by a large meta-analysis in 2024 (15). The intimate link between migraine and PMDD was fortified by previous studies (16, 17), without paying attention to whether the condition is more related to menstrual migraine than non-menstrual type or not. Yet, no significant difference was found between the two groups. The hypo-serotoninergic state, provoked by sudden estrogen withdrawal in the premenstrual period, is implicated in both the affective symptoms of PMDD and the pain mechanisms of migraine (18, 19). This effect may be further amplified in individuals with heightened neurohormonal sensitivity, reflecting increased susceptibility to hormonal fluctuations and their potential influence on migraine severity (20). In agreement with this view, the current study found that the presence of PMDD was associated with an increase of 3.113 points in HIT-6 total score.

The current study showed significantly higher migraine burden among women with menstrual-related migraine in comparison to those with pure menstrual migraine. This can be explained by the significantly higher representation of irregular menses (*p* = 0.046) among those with menstrual-related migraine in comparison to the latter group, which makes the group of menstrual-related migraine prone to marked hormonal fluctuation associated with the irregular menstrual cycles (21). It is worthy to mention that migraine severity was assessed using the HIT-6 score, which evaluates headache severity globally over 4 weeks. That is why it was not possible to separately score the severity of migraine attacks related and unrelated to menses.

Additionally, a significant correlation was found between HIT-6 scores and the utilization of some premenstrual coping strategies, including the scores of Avoiding harm, Awareness and acceptance and Adjusting energy subscales. Comprehensively, the Avoiding harm subscale signifies how a woman deals with the situation by withdrawing from people. On the other hand, the Awareness and Acceptance subscale evaluates the way a woman copes by becoming aware of her physical and psychological changes and recognizing them as a natural part of her experience. Finally, the adjusting energy subscale captures how a woman manages by adjusting her behaviour to control her physical and emotional state, such as emotional outbursts, increased sugar intake, and decreased exercise (11).

However, while the HIT-6 total score showed statistically significant positive correlations with avoiding harm and adjusting energy scores, a significant negative correlation was observed with awareness and acceptance scores. Noticeably, the observed correlations are in a plausible direction.

Accordingly, the current research denotes that higher HIT-6 scores were significantly associated with some maladaptive or dysfunctional premenstrual coping strategies, including social withdrawal, increased sugar intake, and decreased exercise, as denoted by earlier reports (22–24). However, causation cannot be settled because of the cross-sectional design, so we cannot rule out the possibility that higher headache burden leads to increased use of avoidance behaviors. On the other hand, the awareness and acceptance domain is counted as an active coping strategy in the context of chronic pain disorders, which is strongly associated with a better quality of life (25). This involves shifting from trying to eliminate the pain to living a meaningful, valuable life despite its presence. Hence, the present study found that lower awareness and acceptance scores were independently associated with a higher HIT-6 total score.

Taken together, this study imposes the role of psychiatrists to discuss with females with MM and comorbid PMS their psychological readiness and address the potential healthy coping mechanisms to decrease the impact of PMS on their migraine burden. Interestingly, the acceptance and awareness domain is considered a cornerstone in modern (third-wave) Cognitive Behavioral Therapy (CBT), particularly within Acceptance and Commitment Therapy (ACT), which has shown fruitful results in migraine management (26).

Notwithstanding the worthy findings, some limitations must be acknowledged. First, the lack of a healthy control group. Additional research should include representative samples of healthy women without migraine to evaluate potential differences in specific premenstrual coping strategies between the migraine patients and the healthy population based on the psychological background of migraine. Another methodological limitation pertains to the study design. Diary-based prospective studies would be more reliable and informative to reduce the recall bias. Furthermore, differences in group sizes (MM and non-MM) were present, which may have introduced some bias; however, the groups were comparable in baseline demographic and menstrual characteristics, and both groups had sufficient sample sizes to support the study findings. Yet, although the overall sample size was sufficient for the primary comparison between menstrual migraine and non-menstrual migraine, subgroup analyses, particularly those involving PMDD, were underpowered due to the small number of affected participants. This limited the precision of regression estimates and precluded detailed evaluation of PMDD-specific coping strategies. Larger, multicenter studies with expanded recruitment are warranted to validate these findings and provide sufficient power for subgroup analyses. Additionally, the exclusion of patients using migraine preventive medications limits the generalizability of the findings, as the results may not fully represent the full spectrum of migraine sufferers.

## Conclusion

Women with a confirmed diagnosis of PMDD, high PMS symptom severity and lower frequency of using the awareness and acceptance coping strategy are associated with increased migraine burden.

This study suggests that physicians should pay more attention to PMS and PMDD by considering them as part of routine health care for women with migraine, especially those with MM. Finally, it is recommended for interested researchers to conduct a prospective cohort study to plan and implement the most effective coping strategies for this specific female population.

## Data Availability

The original contributions presented in the study are included in the article/supplementary material, further inquiries can be directed to the corresponding author.
